# Predictors of Pre- and Postoperative Quality of Life and Overall Survival in Patients with Non-Small Cell Lung Cancer: A Prospective Study

**DOI:** 10.3390/cancers18040714

**Published:** 2026-02-23

**Authors:** Ryuta Fukai, Tomoki Nishida, Nobuo Yamaguchi, Hideyasu Sugimoto, Tomoya Fukui, Satoshi Noma, Makoto Hibino, Shigeto Horiuchi, Tetsuri Kondo, Keiko Asou, Etsuko Shimizu, Shinichi Teshima, Yumiko Minagawa, Toshitaka Tsukiyama

**Affiliations:** 1 Department of General Thoracic Surgery, Shonan Kamakura General Hospital, 1370-1 Okamoto, Kamakura 247-8533, Kanagawa, Japan; nishida.tomoki.lb@teikyo-u.ac.jp (T.N.); dowarfu@gmail.com (N.Y.); 2 Department of Surgery, Teikyo University School of Medicine, 2-11-1 Kaga, Itabashi-ku 173-0003, Tokyo, Japan; 3 Department of Respiratory Medicine, Fujisawa City Hospital, 2-6-1 Fujisawa, Fujisawa 251-8550, Kanagawa, Japan; hideyasu-sugimoto@live.jp; 4 Department of Respiratory Medicine, Shonan Kamakura General Hospital, 1370-1 Okamoto, Kamakura 247-8533, Kanagawa, Japan; tofukui@med.kitasato-u.ac.jp (T.F.); nomastsh@gmail.com (S.N.); 5 Department of Respiratory Medicine, Shonan Oiso Hospital, 21-1 Gakkyo, Oiso 259-0114, Kanagawa, Japan; m-hibino@ctmc.jp; 6 Department of Respiratory Medicine, Shonan Fujisawa Tokushukai Hospital, 1-5-1 Tsujidokandai, Fujisawa 251-0041, Kanagawa, Japan; shigeto.horiuchi@ctmc.jp (S.H.); tetsuri@ctmc.jp (T.K.); 7 Center for Clinical Research, Shonan Kamakura General Hospital, 1370-1 Okamoto, Kamakura 247-8533, Kanagawa, Japan; k_asou2@shonankamakura.or.jp; 8 Clinical Research Center, Shonan Fujisawa Tokushukai Hospital, 1-5-1 Tsujidokandai, Fujisawa 251-0041, Kanagawa, Japan; etsuko.shimizu@tokushukai.jp; 9 Department of Pathology, Shonan Kamakura General Hospital, 1370-1 Okamoto, Kamakura 247-8533, Kanagawa, Japan; steshima@shonankamakura.or.jp; 10 Department of Radiation Oncology, Shonan Kamakura General Hospital, 1370-1 Okamoto, Kamakura 247-8533, Kanagawa, Japan; miina0913@hotmail.co.jp; 11 Department of Interventional Radiology, Shonan Kamakura General Hospital, 1370-1 Okamoto, Kamakura 247-8533, Kanagawa, Japan; ttsukiyama@shonankamakura.or.jp

**Keywords:** lung cancer, lung cancer surgery, quality of life, prognosis, performance status, Charlson Comorbidity Index

## Abstract

Background/Objectives: Surgery is the most effective treatment for early-stage lung cancer but imposes a greater physical burden than other therapies. We previously identified socioclinical factors associated with reduced perioperative health-related quality of life (HR-QOL) in patients undergoing anatomical pulmonary resection. This study aimed to evaluate the relationship between predictors of HR-QOL and long-term survival. Methods: In this prospective study, 87 patients undergoing anatomical pulmonary resection for non-small cell lung cancer at Shonan Kamakura General Hospital, Japan, were evaluated using the Short Form Health Survey 36. Multivariable analyses identified socioclinical factors associated with physical and mental QOL preoperatively and at 6 months postoperatively. Survival analyses were performed for factors showing differences in multivariable analysis and related trends in univariable analysis of HR-QOL. Results: Preoperatively, lower performance status (PS) and living alone were independently associated with poorer physical QOL (regression coefficient [95% CI]: −10.94 [−14.34–−7.54] and −9.86 [−13.89–−5.82], respectively; both *p* < 0.001) and mental QOL (−9.34 [−13.30–−5.37] and −10.33 [−15.30–−5.35]; both *p* < 0.001). At 6 months postoperatively, smoking cessation within 1 year, lower PS, and living alone predicted worse physical QOL, while smoking cessation and lower PS predicted worse mental QOL. Lower PS and higher comorbidity burden were also adverse prognostic factors for long-term survival (*p* < 0.001 and *p* = 0.015, respectively). Conclusions: Reduced physical activity and greater comorbidity are associated with poorer HR-QOL and survival after lung cancer surgery. These findings highlight the need for careful consideration of surgical indications in patients with these risk factors.

## Simple Summary

Surgery is the most effective treatment for early-stage lung cancer, but it can significantly affect a patient’s physical condition. Therefore, understanding its impact on daily life is crucial for maintaining health status and achieving a favorable long-term prognosis. We conducted a prospective study to investigate socioclinical factors associated with perioperative quality of life and prognosis in patients who underwent anatomical lung resection for lung cancer with curative intent at our institution. Prior to surgery, reduced physical activity and living alone were associated with lower quality of life. At 6 months post-surgery, smoking cessation within 1 year prior to surgery, decreased physical activity, and living alone were significantly associated with reduced quality of life. Patients with reduced physical activity and more comorbidities had significantly worse outcomes. Surgical indications for lung cancer should be carefully considered in patients with reduced physical activity or greater comorbidity.

## 1. Introduction

Lung cancer remains the leading cause of cancer-related mortality worldwide [1], making its treatment a critical global health issue. Approximately 1.8 million deaths each year are attributable to lung cancer [2], and in Japan alone, nearly 75,000 individuals died from the disease in 2023. Surgery is considered the most effective treatment for patients with early-stage lung cancer; however, it imposes a substantial physical burden. In recent years, the average age of patients undergoing lung cancer surgery has increased, with individuals aged 70 years or older accounting for 64.8% of surgical cases in Japan [3]. Older adults frequently have multiple comorbidities and greater frailty than younger individuals, which may increase their vulnerability to surgical stress and postoperative complications [4].

Patient-reported outcomes, including health-related quality of life (HR-QOL), symptoms, and functional status, serve as important indicators alongside recurrence and survival rates [5,6,7], and this relevance has been observed across different disease stages [8]. Previous studies have shown that HR-QOL declines after lung cancer surgery and is influenced by factors such as age, sex, smoking status, comorbid conditions, type of surgery, and receipt of adjuvant treatment [9]. We previously investigated factors contributing to declines in both physical and psychological QOL after lung cancer surgery and reported that living conditions, a high comorbidity burden, more recent smoking, living alone, and reduced physical activity were associated with postoperative HR-QOL deterioration [10,11].

Few studies have prospectively examined prognosis within the same patient cohort in which factors affecting HR-QOL, including preoperative status, were repeatedly assessed. In this study, we prospectively evaluated whether factors associated with reduced QOL at 6 months after surgery influence long-term prognosis following lung cancer surgery.

## 2. Patients and Study Methods

This study included 87 patients who underwent anatomical lung resection for lung cancer with curative intent at our institution between April 2015 and April 2017 and who provided written informed consent. Detailed patient selection criteria have been described in a previous report [10] (Supplementary Figure S1). Participants completed the Short Form Health Survey 36 (SF-36) questionnaire during outpatient visits at baseline and at 1, 3, 6, and 12 months postoperatively.

### 2.1. QOL Assessment

The Japanese version of the SF-36 was used for perioperative QOL assessment [12]. The SF-36 is a self-administered questionnaire consisting of 36 items that assess HR-QOL over the preceding 4 weeks [8]. The questionnaire items are divided into eight subscales, enabling the assessment of both physical and mental quality of life [13]. In this study, physical QOL (P-QOL) was evaluated using four subscales: physical function (PF), role-physical (RP), bodily pain (BP), and general health (GH). Mental QOL (M-QOL) was assessed using four subscales: mental health (MH), role-emotional (RE), social functioning (SF), and vitality (VT). Raw scores were standardized on a scale from 0 to 100, with higher scores indicating better health status. Scores were automatically converted to norm-based scores comparable with the national standard level, which is defined as 50, with a 95% confidence interval used to interpret health status. For both P-QOL and M-QOL, the mean values of the four corresponding subscales were used as each patient’s overall QOL score. For analyses of perioperative changes in individual subscales, mean subscale scores across all patients were calculated at each time point.

### 2.2. Smoking Status

Smoking status was divided into two groups: patients who had stopped smoking within 1 year before surgery and remote or never smokers (i.e., those who had never smoked or had stopped smoking more than 1 year before surgery). Details regarding smoking cessation guidance and verification have been described previously [10].

### 2.3. Performance Status

Preoperative physical activity was assessed using the Eastern Cooperative Oncology Group (ECOG) performance status (PS) scale [14]. Detailed assessment methods have been reported previously [10]. Preoperative PS for each patient was confirmed through review of medical records.

### 2.4. Living Conditions

Patients’ living conditions were classified into two groups: living alone or living with others.

### 2.5. Charlson Comorbidity Index

Preoperative comorbid status was evaluated using the Charlson Comorbidity Index (CCI), applying the modified version proposed by Birim et al. [15].

We prospectively assessed perioperative HR-QOL and examined its relationship with patient characteristics, including age, sex, smoking status, ECOG PS, living conditions, and CCI [10,11]. Based on these findings, we conducted an additional analysis focusing on the association between PS and M-QOL. HR-QOL scores at 6 months postoperatively were selected as the postoperative measure, as most of the eight HR-QOL subscales plateaued between 3 and 6 months after surgery, consistent with previous studies [16,17,18].

### 2.6. Statistical Analysis

For each of the eight SF-36 subscales, a higher QOL score indicates better health. For all patients, the mean scores of the four P-QOL subscales and the four M-QOL subscales were calculated and used to evaluate associations between socioclinical factors and HR-QOL. All statistical analyses were performed using EZR version 1.68, a free statistical software based on R Commander, which is widely used in clinical research [19].

To examine changes in QOL scores across the eight perioperative SF-36 subscales, we applied repeated-measures ANOVA. Paired *t*-tests were used to compare preoperative and postoperative mean HR-QOL scores for all subscales. The Mann–Whitney U test was applied to compare HR-QOL scores between two groups defined by clinical factors (age, sex, smoking status, PS, living conditions, and CCI). Variables showing significant differences on univariable analysis were included in multivariable analysis using multiple regression.

Survival analyses were conducted for factors showing meaningful differences in multivariable analysis and related trends in univariable analysis of HR-QOL. Overall survival was calculated from the date of surgery to either death or the last follow-up, with Kaplan–Meier curves used to estimate survival rates and the log-rank test applied for group comparisons. To account for potential confounding factors influencing survival time, we additionally performed Cox proportional hazards regression analysis on variables that showed significant differences in the log-rank test.

A *p*-value < 0.05 was considered statistically significant.

## 3. Results

Details regarding survey completion rates and response percentages for all patients have been reported previously [10]. Of the 87 patients, 64 (74%) underwent surgery alone, while 23 (26%) received adjuvant therapy within 1 year postoperatively, including platinum-based chemotherapy (*n* = 11, 48%), oral uracil–tegafur (*n* = 10, 44%), radiotherapy (*n* = 1, 4%), and afatinib (*n* = 1, 4%). The median observation period was 85 months (range, 11–121 months). During follow-up, recurrence occurred in 26 of 87 cases (30%), including 14 cases of local recurrence (54%) and 12 cases of distant metastasis (46%). Among these, eight patients received drug therapy (chemotherapy, molecular targeted therapy, or immunotherapy), five received both drug therapy and radiotherapy, and one underwent brain metastasis resection followed by chemotherapy. The median time to recurrence was 23 months (one case with unknown recurrence date), and the median follow-up period after recurrence was 30 months (range, 0–98 months). By December 2025, 21 of 87 patients (24%) had died: 9 from lung cancer, 11 from other causes, and 1 from an unknown cause. The sociodemographic and clinical characteristics of the patients are presented in Table 1. Upon re-examination of the data, discrepancies were noted in patients’ PS compared with previous reports [10], and the data have been revised accordingly.

### 3.1. Association Between Clinical Factors and Preoperative HR-QOL on Univariable Analysis

Univariable analyses were performed to examine associations between clinical factors and preoperative HR-QOL scores. Results are summarized in Table 2. P-QOL scores were significantly lower in patients aged ≥70 years (*p* = 0.016), while M-QOL scores were significantly lower in patients who had quit smoking within 1 year before surgery (*p* = 0.022). Patients with ECOG PS ≥ 1 had significantly lower P-QOL and M-QOL scores compared with those with PS 0 (*p* < 0.001, both). Additionally, patients living alone had significantly lower P-QOL and M-QOL scores compared with those living with others (*p* = 0.0015 and *p* < 0.001, respectively).

### 3.2. Association Between Clinical Factors and Preoperative HR-QOL on Multivariable Analysis

Multivariable analyses were performed separately for P-QOL and M-QOL, including factors that were meaningful in univariable analyses. For P-QOL, ECOG PS ≥ 1 (*p* < 0.001) and living alone (*p* < 0.001) were identified as independent factors of lower preoperative P-QOL scores (Table 3). For M-QOL, the results were similar, with PS ≥ 1 (*p* < 0.001) and living alone (*p* < 0.001) independently associated with lower preoperative M-QOL scores (Table 4).

### 3.3. Perioperative Progress of HR-QOL Scores

**Figure 1:**
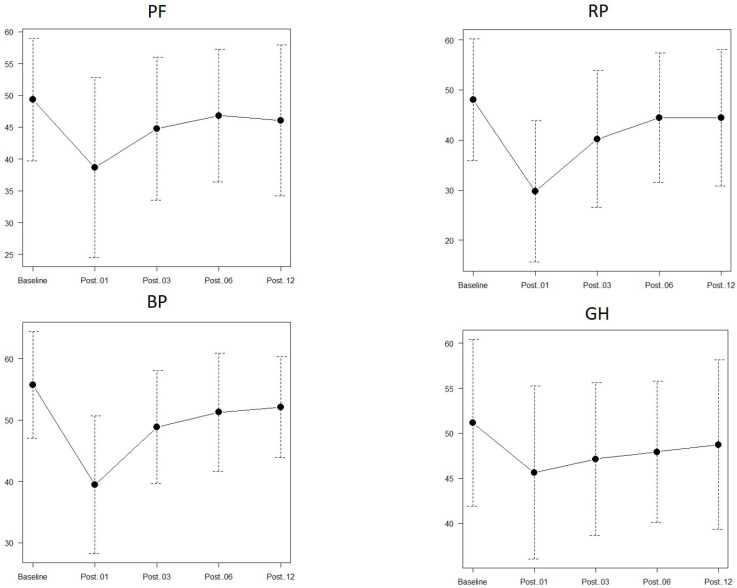
Perioperative progress of the scores of the four physical quality of life subscales. PF, physical function; RP, role-physical; BP, bodily pain; GH, general health; Post.01, 1 month after surgery; Post.03, 3 months after surgery, Post.06, 6 months after surgery; Post.12, 12 months (1 year) after surgery.

P-QOL scores across all four subscales decreased significantly 1 month after surgery compared with preoperative scores (*p* < 0.001) and generally plateaued by 6 months postoperatively (Figure 1). For PF, scores remained significantly lower than preoperative levels even at 1 year postoperatively (*p* = 0.0118). For RP, recovery to levels not significantly different from baseline was achieved by 6 months postoperatively (*p* = 0.348). BP and GH remained significantly lower than baseline at 6 months postoperatively (*p* = 0.0152, *p* = 0.0072, respectively) but recovered to nonsignificant differences at 1 year postoperatively (*p* = 0.0848, *p* = 0.2175, respectively) (Figure 1).

**Figure 2:**
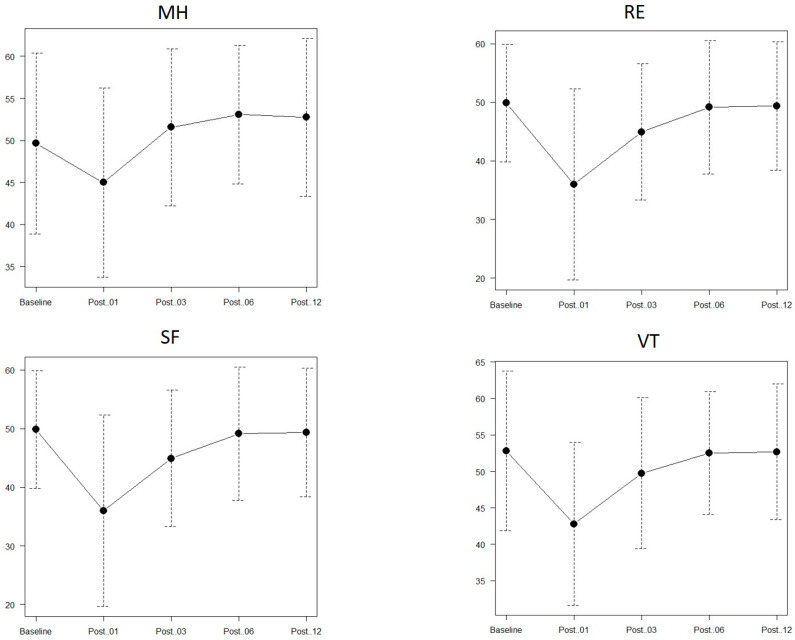
Perioperative progress of the four mental quality of life subscales. MH, mental health; RE, role-emotional; SF, self-functioning; VT, vitality; Post.01, 1 month after surgery; Post.03, 3 months after surgery, Post.06, 6 months after surgery; Post.12, 12 months (1 year) after surgery.

In M-QOL scores, all four subscales decreased significantly one month after surgery compared with preoperative scores (*p* < 0.001) and generally plateaued at 6 months postoperatively. For MH and VT, QOL scores recovered to levels not significantly different from preoperative levels at 3 months postoperatively (*p* = 0.84, *p* = 0.152, respectively). RE and SF returned to baseline at 6 months postoperatively (Figure 2).

### 3.4. Association Between Clinical Factors and Postoperative HR-QOL on Univariable Analysis

Postoperative HR-QOL scores, which plateaued at 6 months after surgery, were compared across patient characteristics. For P-QOL, significantly lower scores were observed in patients who had quit smoking within 1 year preoperatively (*p* = 0.006), had ECOG PS ≥ 1 (*p* < 0.001), or lived alone (*p* = 0.036). For M-QOL, significantly lower scores were observed in patients who had quit smoking within 1 year preoperatively (*p* = 0.048) and in those with PS ≥ 1 (*p* < 0.001). Living alone and higher CCI scores showed a trend toward lower postoperative M-QOL, although these did not reach statistical significance (*p* = 0.058 for both) (Table 5).

### 3.5. Association Between Clinical Factors and Postoperative HR-QOL on Multivariable Analysis

Multivariable analyses were performed separately for P-QOL and M-QOL, including factors that were meaningful in univariable analyses. For P-QOL, quitting smoking within 1 year before surgery (*p* = 0.017), ECOG PS ≥1 (*p* < 0.001), and living alone (*p* = 0.017) were identified as independent predictors of lower postoperative P-QOL scores (Table 6). For M-QOL, quitting smoking within 1 year before surgery (*p* = 0.009) and PS ≥1 (*p* < 0.001) were independent predictors of lower postoperative M-QOL scores (Table 7).

### 3.6. Relationship Between Factors Associated with Postoperative QOL Decline and Prognosis

**Figure 3:**
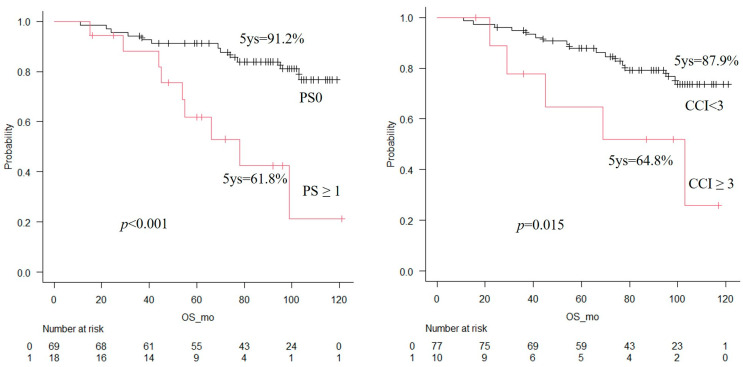
Survival curves by PS and comorbidity. PS, performance status; CCI, Charlson Comorbidity Index; 5ys, 5-year survival rate.

Among socioclinical factors obviously associated with poorer postoperative HR-QOL, lower PS (≥1) and higher comorbidity burden (CCI ≥ 3) were also identified as adverse prognostic factors (*p* < 0.001 and *p* = 0.015, respectively) (Figure 3). In contrast, no significant differences in survival were observed with respect to smoking status (*p* = 0.47) or living conditions (*p* = 0.75).

**Figure 4:**
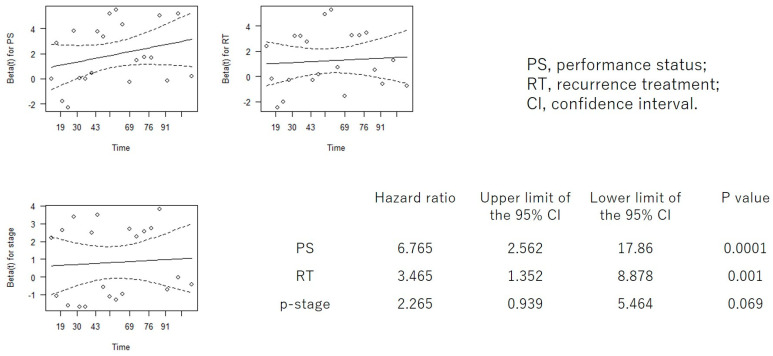
Cox proportional hazards regression analysis for PS, RT, and p-stage. PS, performance status; RT, recurrence treatment; p-stage, pathological stage; CI, confidence interval.

**Figure 5:**
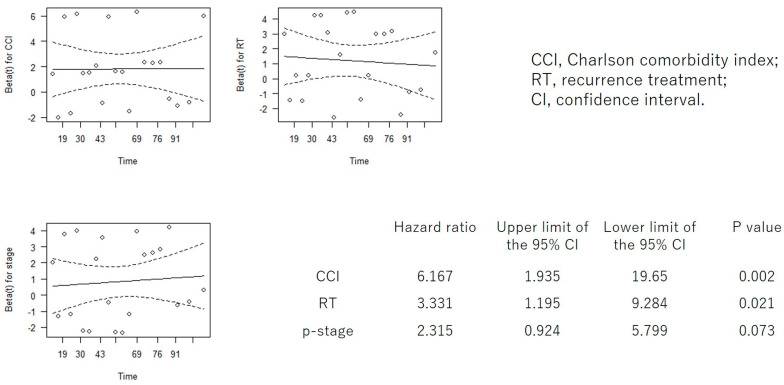
Cox proportional hazards regression analysis for CCI, RT, and p-stage. CCI, Charlson Comorbidity Index; RT, recurrence treatment; p-stage, pathological stage; CI, confidence interval.

In addition to PS and CCI, which showed significant differences in the log-rank test, we performed Cox proportional hazards regression analysis, including factors considered to influence prognosis: age, stage, adjuvant therapy, and recurrence treatment (Supplementary Materials Figure S2). When the Cox hazard regression analysis was repeated using only the three items with the highest hazard ratios (including PS or CCI), both PS and CCI remained significant prognostic factors, each demonstrating the highest hazard ratios (Figure 4 and Figure 5).

## 4. Discussion

Surgery is considered the most effective and potentially curative treatment for patients with early-stage lung cancer; however, it imposes a greater physical burden than other therapeutic modalities. Previous studies have reported that HR-QOL declines for a period after lung cancer surgery [16,18,20]; nevertheless, few studies have examined whether factors associated with HR-QOL also influence prognosis within the same patient cohort. In this prospective study, we investigated factors associated with postoperative HR-QOL decline and evaluated their relationship with long-term prognosis in 87 patients who underwent anatomical pulmonary resection for non-small cell lung cancer at Shonan Kamakura General Hospital, Kanagawa, Japan, using the SF-36. Among socioclinical factors linked to poorer postoperative HR-QOL, lower PS (≥1) and higher comorbidity burden (CCI ≥ 3) were also identified as adverse prognostic factors for survival (*p* < 0.001 and *p* = 0.015, respectively). They were also the most significant prognostic factors with the highest hazard ratios in Cox proportional hazards analysis. Given the increasing age of lung cancer patients, who are often more physically frail and have multiple comorbidities [4,21], these findings provide important information for guiding surgical eligibility and patient management.

PS has been reported to be associated with prognosis in patients with various types of cancer [22,23,24], including those with early-stage lung cancer undergoing surgery [25], as well as patients with advanced-stage disease [26,27]. In our cohort, in which the majority of patients had stage I or II disease, the 5-year survival rate was 91.2% for patients with PS 0, compared with 61.8% for patients with PS ≥ 1. Among patients with PS ≥ 1, nine deaths (50%) occurred during the study period. Of these, eight deaths (88.9%) were due to causes other than lung cancer, and only one death (11.1%) was attributed to lung cancer. These findings suggest that patients with reduced PS may be at higher risk of mortality from non–cancer-related causes. Billé A et al. measured preoperative physical activity over 15 consecutive days in 90 patients and reported that preoperative physical activity levels could help predict postoperative outcomes and stratify the risk of postoperative complications [28]. Moreover, Machado et al. demonstrated in a multicenter randomized controlled trial that preoperative home-based exercise effectively prevents declines in QOL after lung cancer surgery [29]. Accurate preoperative assessment of PS, combined with targeted exercise interventions for patients with lower PS, may help maintain postoperative QOL and improve long-term outcomes following lung cancer surgery.

Charlson et al. developed a comorbidity index for 599 medical patients that accounts for both the number and severity of comorbidities. In a 10-year follow-up of a separate cohort of 685 patients, they reported that cumulative mortality increased with higher comorbidity scores [30]. Birim et al. later demonstrated in 2005 that the CCI is a predictor of long-term postoperative outcomes in patients with non-small cell lung cancer [31]. Their study reported a 5-year survival rate of 52% for all patients, whereas in our cohort, the 5-year survival rate was 64.8% for patients with CCI ≥ 3 and 87.9% for those with CCI < 3. This difference likely reflects advances in lung cancer treatment over the past 20 years and the predominance of early-stage disease in our patient population. Among patients with CCI ≥ 3, five deaths (50%) occurred during the follow-up period: four (80%) from non-cancer causes and one (20%) from lung cancer. Although CCI was not meaningfully associated with perioperative HR-QOL decline in this study, these findings suggest that patients with multiple comorbidities remain at higher risk of death from other causes. Even in early-stage lung cancer, high CCI scores are indicative of poorer long-term prognosis, underscoring the need for careful consideration when determining surgical eligibility in patients with notable comorbidity.

Few studies have investigated factors affecting QOL prior to surgery for early-stage lung cancer, whereas many reports have focused on the impact of lung cancer treatment on HR-QOL. In this study, we found that both reduced PS and living alone were independent predictors of lower P-QOL and M-QOL in multivariable analyses prior to surgery. PS is a reliable indicator of a patient’s overall condition and is a critical factor in determining cancer treatment options (surgery, radiation therapy, or systemic therapy) and the intensity of palliative care [32]. Significant differences in preoperative HR-QOL were observed between patients with PS 0 and those with PS ≥ 1 (*p* < 0.001), and these pre-existing differences may also influence postoperative prognosis. Living alone was associated with significantly lower preoperative P-QOL (*p* = 0.0015) and M-QOL (*p* < 0.001). Previous studies suggest that cohabitation with family can promote healthy behaviors through direct and indirect social control [33]. Jeong et al. reported that men living alone had a 1.80-fold higher risk of physical inactivity, compared with those living with others [34]. Our previous studies also demonstrated that smoking and decreased physical activity are linked to lower HR-QOL [10,11]. Together, these findings suggest that lifestyle factors such as smoking and reduced activity may contribute to decreased HR-QOL among individuals living alone.

At 6 months after lung cancer surgery, when postoperative HR-QOL scores plateaued, recent smoking cessation, lower PS (≥1), and living alone were independently associated with apparent declines in HR-QOL in multivariable analyses. Furthermore, decreased PS (*p* < 0.001) and higher comorbidity burden (*p* = 0.0015) were significant prognostic factors following lung cancer surgery. Traditionally, surgical invasiveness has been evaluated based on factors such as the surgical approach (open thoracotomy vs. minimally invasive surgery), blood loss, and postoperative pain. However, it is also critically important to assess the extent and duration of surgery’s impact on a patient’s QOL. Physical function in patients with non-small cell lung cancer often deteriorates following lung surgery, regardless of whether (neo-)adjuvant treatment is administered during the first year after the procedure [35]. These physical functions include peripheral muscle strength (e.g., quadriceps and grip strength), respiratory muscle function (e.g., maximal inspiratory and expiratory pressures), functional exercise capacity (e.g., 6 min walk distance and stair-climbing test), maximal exercise capacity (e.g., maximal oxygen consumption), and overall physical activity (e.g., total daily steps and sedentary time). Lung cancer surgery has been shown to impair these functions, with similar effects observed after both minimally invasive surgery (VATS or RATS) and open thoracotomy [36]. Surgeons should carefully evaluate whether surgery represents the optimal treatment option for each patient, considering not only postoperatively QOL but also long-term prognosis and weighing it against alternative therapies.

This study has several limitations. First, it was conducted at a single institution with a relatively small sample size, although the study design was prospective. Second, the results were based on patients’ self-reported assessments, which are subjective and not objective evaluations. Nonetheless, because lung cancer treatment is intended for the patient’s benefit, the patient’s perspective is considered highly relevant. Third, we did not fully account for the potential influence of postoperative adjuvant therapy on HR-QOL and prognosis, although the majority of patients (84%) underwent surgery alone. Of the 23 patients who received adjuvant therapies, 10 (44%) received oral uracil–tegafur, which has been reported not to deteriorate overall HR-QOL during adjuvant chemotherapy in patients with colorectal cancer [36]. However, because oral uracil–tegafur has demonstrated efficacy equivalent to intravenous chemotherapy in postoperative adjuvant therapy, it is possible that improvements in prognosis associated with adjuvant therapy may have influenced our results [37]. Therefore, in this study, oral uracil–tegafur is also considered to have contributed to a certain degree of improvement in prognosis. Fourth, we did not account for the impact of treatment for lung cancer recurrence. Patients who experienced recurrence received drug therapy, radiation therapy, or surgery, all of which likely influenced both QOL and prognosis. Although we did not analyze the effects of these individual treatments on HR-QOL or survival, our results reflect real-world clinical outcomes, which supports the relevance of our findings. Fifth, not all patients completed the self-rated questionnaire, and because it was administered during outpatient visits, it is possible that the hospital setting influenced their responses.

Despite these limitations, we believe that our prospective study provides valuable information for clinicians managing lung cancer. Preoperative assessment of HR-QOL allows prediction of postoperative QOL decline and long-term prognosis, which can guide the selection and planning of treatment strategies tailored to each patient’s condition.

## 5. Conclusions

Lower PS (≥1) and living alone (*p* < 0.001, both) were independently associated with significantly worse HR-QOL in the preoperative setting. At 6 months postoperatively, more recent smoking cessation (within 1 year before surgery) and PS ≥ 1 were independently associated with worse overall HR-QOL, while living alone was independently associated with worse P-QOL.

Even after 1 year, and despite the predominance of less invasive procedures such as thoracoscopic surgery, patients with lung cancer experienced reductions in P-QOL postoperatively, likely reflecting the combined effects of surgery and adjuvant therapies.

Among socioclinical factors linked to poorer postoperative HR-QOL, lower PS (≥1) and higher comorbidity burden (CCI ≥ 3) were also associated with prognosis. In these patients, non-cancer-related factors may contribute substantially to mortality, highlighting the need for careful consideration when determining surgical eligibility.

## Figures and Tables

**Table 1:** Sociodemographic and clinical characteristics of patients (n = 87).

Characteristic	Number (%)
Age (years)	
Mean ± SD	69.7 ± 8.5
Range	48–83
Sex	
Male	41 (47.1)
Female	46 (52.9)
Smoking status	
Stopped within 1 year before surgery	17 (19.5)
Others	70 (80.5)
Performance status	
0	69 (79.3)
1–2	18 (20.7)
Living conditions	
Living alone	10 (11.5)
Living with somebody	77 (88.5)
Charlson Comorbidity Index	
<3	77 (88.5)
≥3	10 (11.5)
Surgical approach	
Thoracoscopic	74 (85.1)
Thoracotomy	13 (14.9)
Procedure	
Segmentectomy	5 (5.75)
Lobectomy	79 (90.8)
Bilobectomy	2 (2.3)
Pneumonectomy	1 (1.15)
Histology	
Adenocarcinoma	73 (83.9)
Squamous cell carcinoma	12 (13.8)
Adenosquamous	1 (1.15)
Carcinoid	1 (1.15)
p-stage	
IA	35 (40.2)
IB	27 (31.0)
IIA	7 (8.0)
IIB	12 (13.8)
IIIA	6 (7.0)

SD, standard deviation.

**Table 2:** Association between clinical factors and preoperative HR-QOL on univariable analysis.

Variables	No. of Patients	P-QOL Score Median (Min–Max)	*p*-Value	M-QOL Score Median (Min–Max)	*p*-Value
Age (years)			0.016		0.88
<70	35	54.8 (35.8–60.9)		51.4 (24.1–61.9)	
≥70	52	50.9 (18.2–60.2)		51.5 (20.3–61.9)	
Sex			0.28		0.33
Male	41	52.0 (18.2–60.0)		52.7 (20.3–61.9)	
Female	46	53.1 (31.1–60.9)		51.2 (24.1–61.9)	
Smoking status			0.051		0.022
Stopped within 1 year before surgery	17	49.2 (18.2–57.3)		45.8 (20.3–60.5)	
Remote or never smokers	70	51.5 ± 7.1 (31.1–60.9)		52.8 (24.1–61.9)	
Performance status			<0.001		<0.001
0	69	54.0 (34.7–60.9)		53.1 (24.1–61.9)	
1 or 2	18	44.8 (18.2–55.3)		45.6 (20.3–57.3)	
Living conditions			0.0015		<0.001
Living alone	10	43.3 (18.2–54.0)		41.1 (20.3–52.9)	
Living with somebody	77	53.5 (30.8–60.9)		52.7 (24.1–61.9)	
Charlson Comorbidity Index			0.1		0.09
<3	77	52.4 (30.8–60.9)		51.2 (24.1–61.9)	
≥3	10	53.4 (18.2–60.0)		58.1 (20.3–61.9)	

P-QOL, physical quality of life; M-QOL, mental quality of life; Min, minimum; Max, maximum.

**Table 3:** Association between clinical factors and preoperative physical quality of life in multivariable analysis.

Variables	Regression Coefficient (95% CI)	*p*-Value
Age (≥70 years or not)	−0.59 (−3.41–2.22)	0.68
Performance status (≥1 or not)	−10.94 (−14.34–−7.54)	<0.001
Living conditions (Living alone or not)	−9.86 (−13.89–−5.82)	<0.001

CI, confidence interval.

**Table 4:** Association between clinical factors and preoperative mental quality of life in multivariable analysis.

Variables	Regression Coefficient (95% CI)	*p*-Value
Smoking status (Stopped smoking within 1 year or not)	−3.22 (−7.29–0.85)	0.12
Performance status (≥1 or not)	−9.34 (−13.30–−5.37)	<0.001
Living conditions (Living alone or not)	−10.33 (−15.30–−5.35)	<0.001

CI, confidence interval.

**Table 5:** Association between clinical factors and postoperative physical and mental quality of life scores on univariable analysis.

Variables	No. of Patients	P-QOL Score Median (Min–Max)	*p*-Value	M-QOL Score Median (Min–Max)	*p*-Value
Age (years)			0.36		0.91
<70	35	49.5 (15.0–57.5)		51.9 (20.1–61.9)	
≧70	52	48.1 (22.4–59.3)		52.5 (21.6–61.2)	
Sex			0.93		0.76
Male	41	48.6 (15.0–57.5)		51.4(20.1–61.2)	
Female	46	48.8 (30.7–59.3)		53.2 (21.6–61.9)	
Smoking status			0.006		0.048
Stopped within 1 year before surgery	17	43.6 (15.0–51.0)		47.2 (20.1–58.8)	
Remote or never smokers	70	50.2 (30.7–59.3)		53.2 (21.6–61.9)	
Performance status			<0.001		<0.001
0	69	50.4 (30.7–59.3)		53.5 (21.6–61.9)	
1 or 2	18	42.0 (15.0–50.4)		44.3 (20.1–54.5)	
Living conditions			0.036		0.058
Living alone	10	44.3 (22.4–55.7)		48.8 (21.6–56.6)	
Living with somebody	77	50.0 (15.0–59.3)		52.8 (20.1–61.9)	
Charlson Comorbidity Index			0.1		0.058
<3	77	49.5 (30.7–59.3)		52.8 (21.6–61.9)	
≧3	10	41.6 (15.0–56.2)		41.6 (20.1–59.6)	

P-QOL, physical quality of life; M-QOL, mental quality of life; Min, minimum; Max, maximum.

**Table 6:** Association between clinical factors and postoperative physical quality of life in multivariable analysis.

Variables	Regression Coefficient (95% CI)	*p*-Value
Smoking status (Stopped smoking within 1 year or not)	−5.04 (−9.12–−0.95)	0.017
Performance status (≥1 or not)	−9.65 (−13.60–−5.70)	<0.001
Living conditions (Living alone or not)	−5.67 (−10.27–−1.07)	0.017

CI, confidence interval.

**Table 7:** Association between clinical factors and postoperative mental quality of life in multivariable analysis.

Variables	Regression Coefficient (95% CI)	*p*-Value
Smoking status (Stopped smoking within 1 year or not)	−5.64 (−9.83–−1.45)	0.009
Performance status (≥1 or not)	−9.56 (−13.64–−5.48)	<0.001

CI, confidence interval.

## Data Availability

The data presented in this study are available upon request from the corresponding author because they contain the patients’ individual medical treatment details, progress, and questionnaire responses.

## Supplementary Materials

The following supporting information can be downloaded at https://www.mdpi.com/article/10.3390/cancers18040714/s1. Figure S1: Patient Selection Flowchart, Figure S2: Cox proportional hazards regression analysis including factors considered to influence prognosis: age, stage, adjuvant therapy, and recurrence treatment.

## Abbreviations

The following abbreviations are used in this manuscript:

BP	Bodily pain
CCI	Charlson Comorbidity Index
GH	General health
MH	Mental health
NSCLC	Non-small cell lung cancer
PF	Physical function
PS	Performance status
QOL	Quality of life
RE	Role-emotional
RP	Role-physical
SF	Social functioning

## References

[1] Smolarz B., Łukasiewicz H., Samulak D., Piekarska E., Kołaciński R., Romanowicz H. (2025). Lung cancer—Epidemiology, pathogenesis, treatment and molecular aspect (review of literature). Int. J. Mol. Sci..

[2] Bray F., Laversanne M., Sung H., Ferlay J., Siegel R.L., Soerjomataram I., Jemal A. (2024). Global cancer statistics 2022: GLOBOCAN estimates of incidence and mortality worldwide for 36 cancers in 185 countries. CA Cancer J. Clin..

[3] Yoshimura N., Sato Y., Takeuchi H., Abe T., Endo S., Hirata Y., Ishida M., Iwata H., Kamei T., Committee for Scientific Affairs, The Japanese Association for Thoracic Surgery (2024). Thoracic and cardiovascular surgeries in Japan during 2021: Annual report by the Japanese Association for Thoracic Surgery. Gen. Thorac. Cardiovasc. Surg..

[4] Panagopoulos N., Grapatsas K., Leivaditis V., Galanis M., Dougenis D. (2023). Are extensive open lung resections for elderly patients with lung cancer justified?. Curr. Oncol..

[5] Bdira B.B., Gargouri I., Benzarti W., Belajouza S., Knaz A., Abdelghani A., Garrouch A., Benzarti M., Hayouni A., Aissa S. (2022). Prognostic value of quality of life (QoL) assessment among Tunisian lung cancer patients. Tunis. Med..

[6] Liu J., Ma Y., Gao R., Liu X., Wang Y., Yu J., Zhan J., Huang Y., Zhang L. (2022). Prognostic effects of health-related quality of life at baseline and early change in health-related quality of life on response to treatment and survival in patients with advanced lung cancer: A prospective observational study in China. BMJ Open.

[7] Trejo M.J., Bell M.L., Dhillon H.M., Vardy J.L. (2020). Baseline quality of life is associated with survival among people with advanced lung cancer. J. Psychosoc. Oncol..

[8] Lemonnier I., Guillemin F., Arveux P., Clément-Duchêne C., Velten M., Woronoff-Lemsi M.C., Jolly D., Baumann C. (2014). Quality of life after the initial treatments of non-small cell lung cancer: A persistent predictor for patients’ survival. Health Qual. Life Outcomes.

[9] Poghosyan H., Sheldon L.K., Leveille S.G., Cooley M.E. (2013). Health-related quality of life after surgical treatment in patients with non-small cell lung cancer. Lung Cancer.

[10] Fukai R., Nishida T., Sugimoto H., Hibino M., Horiuchi S., Kondo H., Teshima S., Hirata M., Asou K., Shimizu E. (2024). Perioperative evaluation of the physical quality of life of patients with non-small cell lung cancer: A prospective study. Cancers.

[11] Fukai R., Nishida T., Igarashi Y., Murata T., Sunoh Y., Miyake K., Isogai N., Shimoyama R., Kawachi J., Kashiwagi H. (2021). Perioperative progress and predictor of the postoperative mental quality of life for lung cancer patients. Br. J. Cancer Res..

[12] Fukuhara S., Bito S., Green J., Hsiao A., Kurokawa K. (1998). Translation, adaptation, and validation of the SF-36 health survey for use in Japan. J. Clin. Epidemiol..

[13] McHorney C.A., Ware J.E., Lu J.F., Sherbourne C.D. (1994). The MOS 36-item short-form health survey (SF-36): III. Tests of data quality, scaling assumptions, and reliability across diverse patient groups. Med. Care.

[14] Oken M.M., Creech R.H., Tormey D.C., Horton J., Davis T.E., McFadden E.T., Carbone P.P. (1982). Toxicity and response criteria of the Eastern Cooperative Oncology Group. Am. J. Clin. Oncol..

[15] Birim O., Maat A.P.W.M., Kappetein A.P., van Meerbeeck J.P., Damhuis R.A.M., Bogers A.J.J.C. (2003). Validation of the Charlson comorbidity index in patients with operated primary non-small cell lung cancer. Eur. J. Cardiothorac. Surg..

[16] Win T., Sharples L., Wells F.C., Ritchie A.J., Munday H., Laroche C.M. (2005). Effect of lung cancer surgery on quality of life. Thorax.

[17] Balduyck B., Hendriks J., Lauwers P., Nia P.S., Van Schil P. (2009). Quality of life evolution after lung cancer surgery in septuagenarians: A prospective study. Eur. J. Cardiothorac. Surg..

[18] Brunelli A., Socci L., Refai M., Salati M., Xiumé F., Sabbatini A. (2007). Quality of life before and after major lung resection for lung cancer: A prospective follow-up analysis. Ann. Thorac. Surg..

[19] Kanda Y. (2013). Investigation of the freely available easy-to-use software ‘EZR’ for medical statistics. Bone Marrow Transplant..

[20] Pompilli C., Brunelli A., Xiumé F., Refai M., Salati M., Sabbatini A. (2011). Predictors of postoperative decline in quality of life after major lung resections. Eur. J. Cardiothorac. Surg..

[21] Christensen N.L., Gouliaev A., McPhail S., Lyratzopoulos G., Rasmussen T.R., Jensen H. (2024). Lung cancer among the elderly in Denmark: A comprehensive population-based cohort study. Lung Cancer.

[22] Tas F., Sen F., Odabas H., Kilic L., Keskin S., Yildiz I. (2013). Performance status of patients is the major prognostic factor at all stages of pancreatic cancer. Int. J. Clin. Oncol..

[23] Colloca G.A., Venturino A. (2024). Prognostic effect of performance status on outcomes of patients with colorectal cancer receiving first-line chemotherapy: A meta-analysis. J. Gastrointest. Cancer.

[24] Evers P.D., Logan J.E., Sills V., Chin A.I. (2014). Karnofsky performance status predicts overall survival, cancer-specific survival, and progression-free survival following radical cystectomy for urothelial carcinoma. World J. Urol..

[25] Kawaguchi Y., Hanaoka J., Oshio Y., Hashimoto M., Igarashi T., Kataoka Y., Kaku R., Namura Y., Akazawa A. (2017). Decrease in performance status after lobectomy mean poor prognosis in elderly lung cancer patients. J. Thorac. Dis..

[26] Chang C.Y., Chen C.Y., Chang S.C., Lai Y.C., Wei Y.F. (2021). Efficacy and prognosis of first-line EGFR-tyrosine kinase inhibitor treatment in older adults including poor performance status patients with EGFR-mutated non-small-cell lung cancer. Cancer Manag. Res..

[27] Olszyna-Serementa M., Zaborowska-Szmit M., Szmit S., Jaśkiewicz P., Zajda K., Krzakowski M., Kowalski D.M. (2023). Performance-status deterioration during sequential chemo-radiotherapy as a predictive factor in locally advanced non-small cell lung cancer. Curr. Oncol..

[28] Billé A., Buxton J., Viviano A., Gammon D., Veres L., Routledge T., Harrison-Phipps K., Dixon A., Minetto M.A. (2021). Preoperative physical activity predicts surgical outcomes following lung cancer resection. Integr. Cancer Ther..

[29] Machado P., Pimenta S., Garcia A.L., Nogueira T., Silva S., Dos Santos C.L., Martins M.V., Canha A., Oliveiros B., Martins R.A. (2024). Effect of preoperative home-based exercise training on quality of life after lung cancer surgery: A multicenter randomized controlled trial. Ann. Surg. Oncol..

[30] Charlson M.E., Pompei P., Ales K.L., MacKenzie C.R. (1987). A new method of classifying prognostic comorbidity in longitudinal studies: Development and validation. J. Chron. Dis..

[31] Birim O., Kappetein A.P., Bogers A.J. (2005). Charlson comorbidity index as a predictor of long-term outcome after surgery for nonsmall cell lung cancer. Eur. J. Cardiothorac. Surg..

[32] Sok M., Zavrl M., Greif B., Srpčič M. (2019). Objective assessment of WHO/ECOG performance status. Support Care Cancer.

[33] Umberson D. (1987). Family status and health behaviors: Social control as a dimension of social integration. J. Health Soc. Behav..

[34] Jeong S., Cho S.I. (2017). Effects of living alone versus with others and of housemate type on smoking, drinking, dietary habits, and physical activity among elderly people. Epidemiol. Health.

[35] Haesevoets S., Arents E., Cops D., Quadflieg K., Criel M., Ruttens D., Ruttens M., Stevens D., Surmont V., Demeyer H. (2025). The impact of lung surgery, with or without (neo-)adjuvant therapy, on physical functioning in patients with nonsmall cell lung cancer: A scoping review. Eur. Respir. Rev..

[36] Tsunoda A., Nakao K., Watanabe M., Matsui N., Tsunoda Y. (2010). Health-related quality of life in patients with colorectal cancer who receive oral uracil and tegafur plus leucovorin. Jpn. J. Clin. Oncol..

[37] Liang S.K., Wu C.W., Chang C.I., Keng L.T., Lee M.R., Wang J.Y., Ko J.C., Liao W.Y., Chen K.Y., Ho C.C. (2023). Oral uracil–tegafur compared with intravenous chemotherapy as adjuvant therapy for resected early-stage non-small cell lung cancer patients. Cancer Med..

