# Supplementary material for: Predictors of Pre- and Postoperative Quality of Life and Overall Survival in Patients with Non-Small Cell Lung Cancer: A Prospective Study
Source: Cancers (Basel). 2026 Feb 23;18(4):714. doi: 10.3390/cancers18040714 (PMC12938989; doi:10.3390/cancers18040714)
Supplement: Supplementary file 1 [file cancers-18-00714-s001.zip › cancers-4102082-supplementary.pdf]

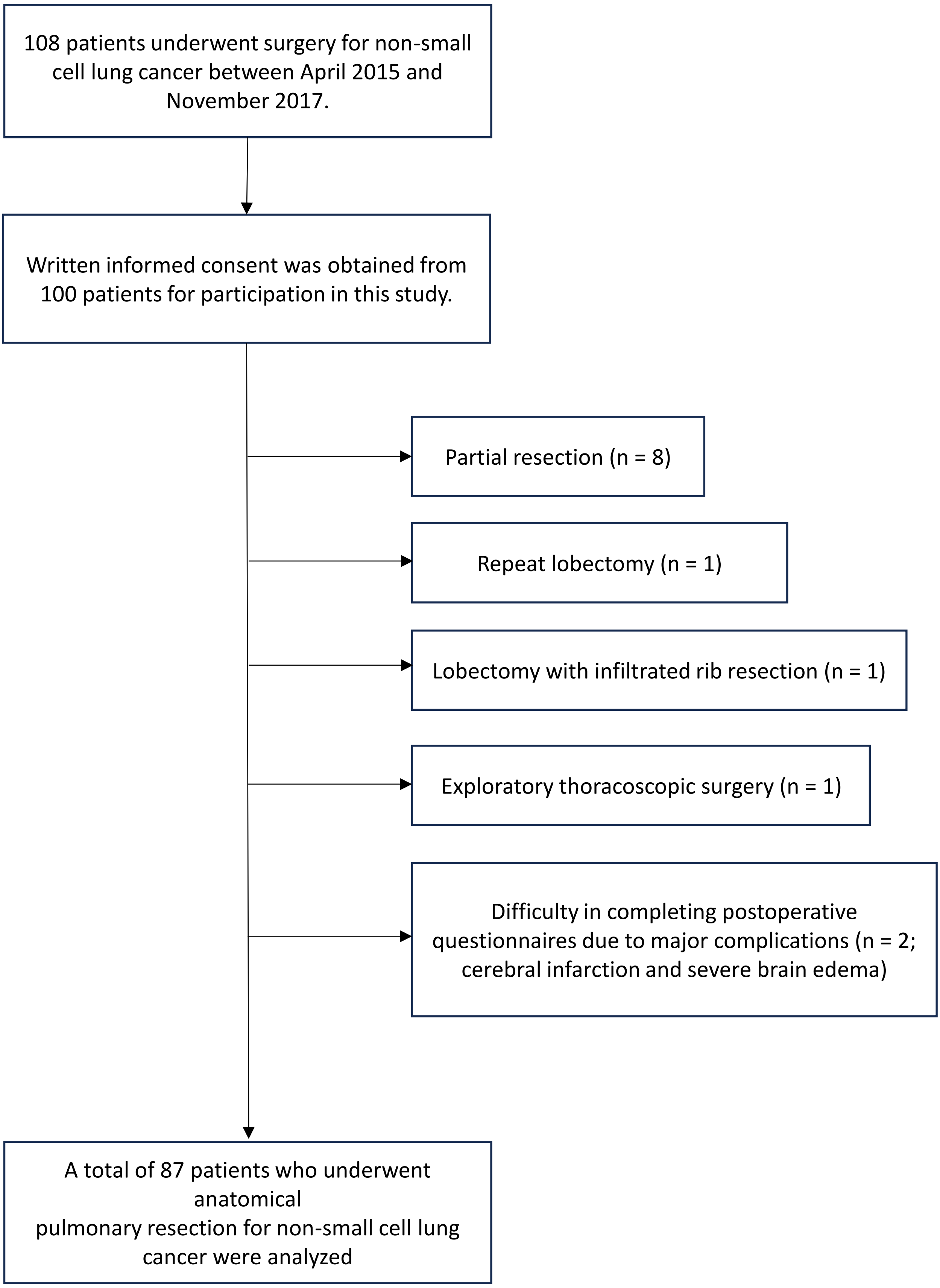

Supplementary material Figure S1. Patient selection flowchart.

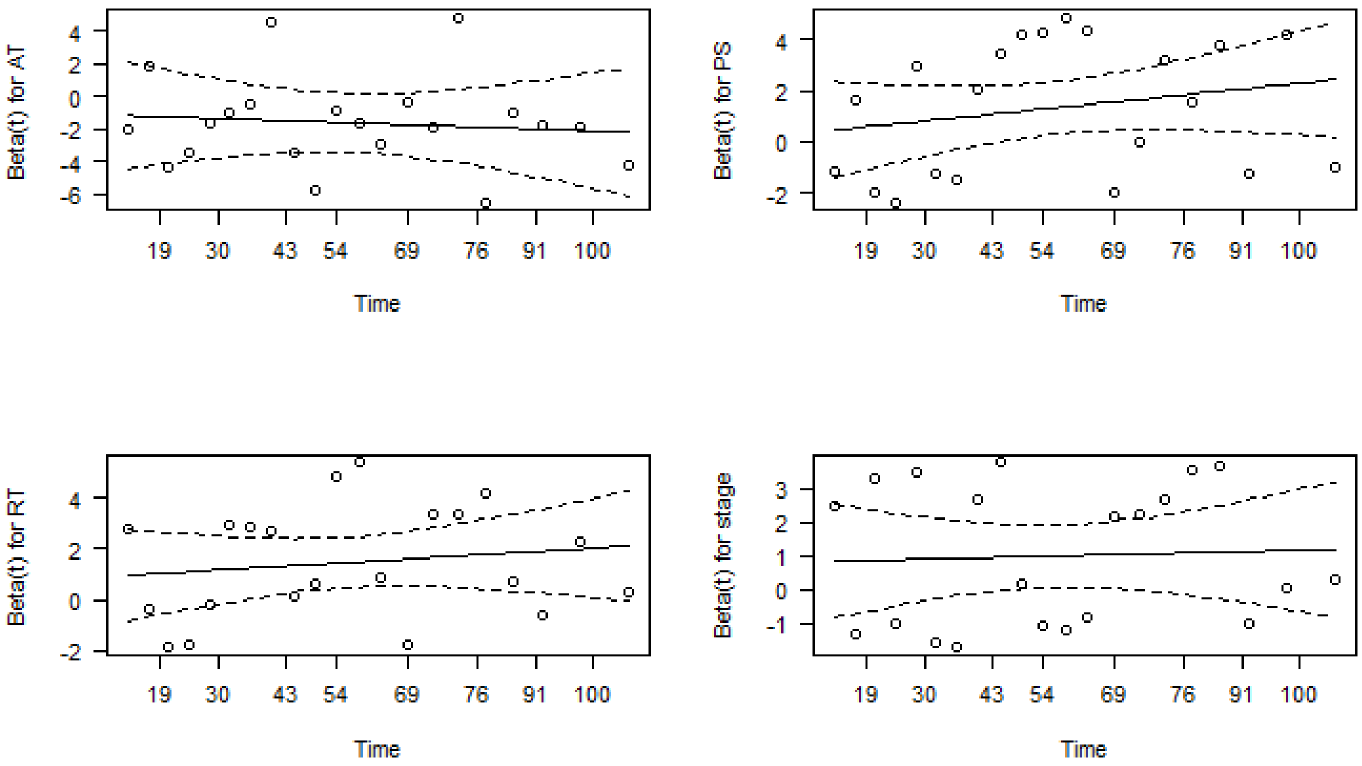

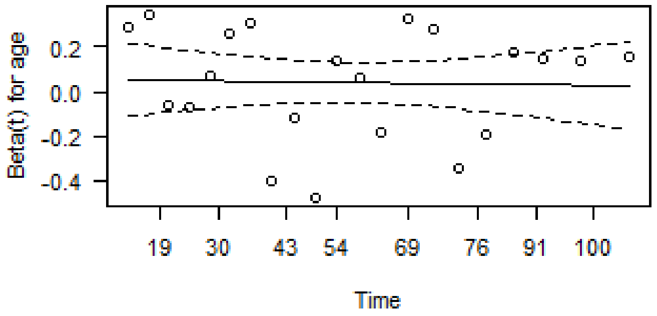

AT, adjuvant therapy;
PS, performance status;
RT, recurrence treatment;
P-stage, pathological stage.

	Hazard ratio	Lower limit of the 95% CI	Upper limit of the 95% CI	P value
AT	0.201	0.034	1.172	0.075
PS	3.785	1.373	10.43	0.01
RT	4.339	1.635	11.51	0.003
p-stage	2.697	1.093	6.656	0.031
age	1.042	0.954	1.137	0.36

CI, confidence interval

Supplementary material Figure S2a. Cox proportional hazards regression analysis for adjuvant therapy, performance status, recurrence treatment, p-stage, and age.

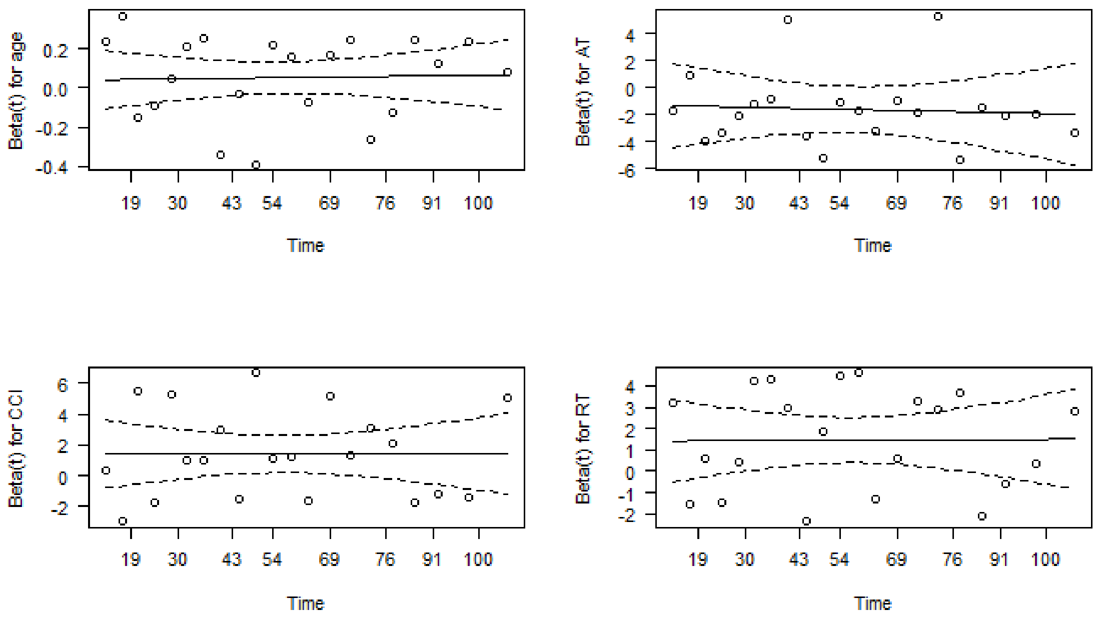

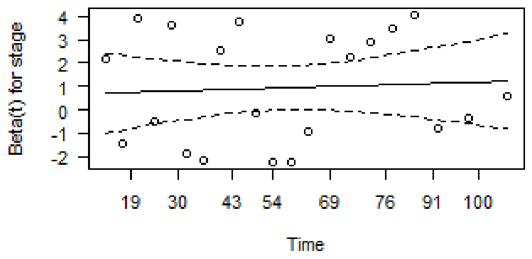

AT, adjuvant therapy;
CCI, Charlson comorbidity index;
RT, recurrence treatment;
P-stage, pathological stage.

	Hazard ratio	Lower limit of the 95% CI	Upper limit of the 95% CI	P value
age	1.053	0.972	1.14	0.204
AT	0.194	0.036	1.034	0.055
CCI	4.062	1.255	13.15	0.019
RT	4.309	1.523	12.19	0.006
p-stage	2.553	1.016	6.42	0.046

CI, confidence interval

Supplementary material Figure S2b. Cox proportional hazards regression analysis for age, adjuvant therapy, Charlson comorbidity index, recurrence treatment, p-stage.
